# Analyses of the Root-Knot Nematode (*Meloidogyne graminicola*) Transcriptome during Host Infection Highlight Specific Gene Expression Profiling in Resistant Rice Plants

**DOI:** 10.3390/pathogens9080644

**Published:** 2020-08-08

**Authors:** Anne-Sophie Petitot, Alexis Dereeper, Corinne Da Silva, Julie Guy, Diana Fernandez

**Affiliations:** 1 IRD, Cirad, Univ Montpellier, IPME, 911 Avenue Agropolis, BP 64501, CEDEX 5, 34394 Montpellier, France; alexis.dereeper@ird.fr (A.D.); diana.fernandez@ird.fr (D.F.); 2 Génomique Métabolique, Genoscope, Institut François Jacob, CEA, CNRS, Univ Evry, Université Paris-Saclay, 91057 Evry, France; dasilva@genoscope.cns.fr (C.D.S.); jguy@genoscope.cns.fr (J.G.)

**Keywords:** plant-parasitic nematode, dual RNA-Seq, resistance, virulence effector, cell wall-degrading enzymes, cuticle, neuropeptide, venom allergen-like protein

## Abstract

The plant-parasitic nematode *Meloidogyne graminicola* causes considerable damages to rice (*Oryza sativa*) culture. Resistance to *M. graminicola* in the related species *Oryza glaberrima* reduces root penetration by juveniles and stops further nematode development. *M. graminicola* genes expressed during *O. sativa* infection were previously characterized but no information is available about the molecular dialogue established with a resistant plant. We compared the *M. graminicola* transcriptomes of stage-two juveniles (J2s) before and during infection of susceptible or resistant rice. Among 36,121 *M. graminicola* genes surveyed, 367 were differentially expressed during infection of resistant or susceptible plants. Genes encoding cell wall-degrading enzymes, peptidases and neuropeptides were expressed for a longer time in resistant plants compared to susceptible plants. Conversely, genes related to nematode development were not activated in the resistant host. The majority of *M. graminicola* effector genes had similar expression patterns, whatever the host genotype. However, two venom allergen-like protein (VAP)-encoding genes were specifically induced in resistant plants and *Mg-VAP1* silencing in J2s reduced their ability to colonize roots. This study highlighted that *M. graminicola* adapts its gene expression to the host susceptibility. Further investigation is required to assess the role of *Mg-VAPs* in the rice–nematode interaction.

## 1. Introduction

Root-knot nematodes (RKN) (*Meloidogyne* species) are obligate sedentary parasites that cause considerable damage to crops by developing into plant roots. The rice nematode *Meloidogyne graminicola* is prevalent across Asian and Latin American paddy rice (*Oryza sativa* L.) fields [1,2], and was recently reported in Italy [3]. Severe infections result in stunted root systems, which ultimately result in poor growth and crop production with substantial yield losses. *M. graminicola*-infected rice plants have characteristic swollen and hooked galls at root tips. RKN establish a feeding site in galls by inducing the differentiation of host cells into hypertrophied, multinucleate and metabolically active cells, named giant cells. This feeding structure serves as a constant food source to the nematode, allowing its development into a reproductive female, and completing its life cycle inside the host root. In rice *O. sativa* Nipponbare plants, giant cells are visible as soon as two days post-infection (dpi) and egg-laying females can be observed from 18 to 22 dpi [4].

A main feature of plant-parasitic nematodes is the presence of a protractible stylet that serves to withdraw nutriments from the giant cells as well as to release molecules, including virulence effectors, in the apoplast or directly into the host cells [5,6]. Effectors are molecules secreted by pathogens into the plant tissues to facilitate infection, by reprograming the host metabolism, or by preventing the execution of plant defense responses. Sedentary nematodes mainly release effectors produced in their esophageal glands, through their stylet into host tissues, although other secretory organs may play a role in the parasitic process [7]. Transcriptomic, proteomic and comparative genomic approaches identified large repertoires of effector gene families in plant-parasitic nematodes [8]. In the rice–*M. graminicola*-compatible interaction, nematode effectors were identified from comprehensive transcriptomic analyses of pre-parasitic J2s and rice infected tissues [9,10], spanning from J2 penetration to development into young adult females (from 2 to 16 dpi) [10]. Over the past years, novel *M. graminicola* effectors playing a role in nematode parasitism were functionally characterized, including pioneer genes [11,12,13], a C-type lectin [14], and a protein disulfide isomerase (PDI) [15]. Several RKN effectors characterized so far on tomato or *Arabidopsis thaliana* are able to interact with host proteins and consequently can interfere with cellular processes within the plant cell or in the apoplast to facilitate nematode infection (for review [6]). In addition, some *M. graminicola* effectors are able to suppress the induced hypersensitive response (HR) in *Nicotiana benthamiana* transient expression assays or reactive oxygen species (ROS) production and may contribute to the protection of nematodes from oxidative damage.

Effector proteins could play a role in suppressing host immunity to allow for successful infection of susceptible plants. However, we have only scarce knowledge about RKN genes that are involved in the infection of resistant hosts. Are there specific effectors produced in response to host defense reactions? Does the nematode adapt its gene expression to different host genotypes? Studies focusing on the secretome of *Meloidogyne incognita* interacting with a resistant host genotype evidenced few changes in nematode gene expression, associated to degrading enzymes, antioxidant, autophagy and cytoskeleton-related processes [16,17].

The aim of this study was to identify the nematode genes expressed in resistant plants and highlight the major changes occurring in their pattern of expression as compared to susceptible plants. In rice, genetic resistance to *M. graminicola* has been found only in a few *O. sativa* varieties [18,19,20] and in some rice relative species, including the African rice *Oryza glaberrima* [21,22] and the South-American wild rice *Oryza glumaepatula* [23]. Root infection by the nematode quickly stops in some rice accessions by a HR-like reaction at the penetration site [20,23], or feeding sites degenerate before the J2s developed into adults, as we observed in *O. glaberrima* TOG5681, for instance [22].

*M. graminicola* transcriptomic responses to plant defenses were established during the first 8 days of the incompatible interaction between the nematode and *O. glaberrima* TOG5681-resistant plants and compared to the dataset produced during the compatible interaction with the *O. sativa* Nipponbare plants and in pre-parasitic J2s [10]. In addition, the *M. graminicola* transcriptome annotation published in Petitot et al. [10] was improved here by novel homology searches against the recently published *M. graminicola* genome [24] and the last released version of the *M. incognita* genome [25]. This study revealed important molecular pathways and candidate effectors genes deregulated in *M. graminicola* infecting resistant plants. To our knowledge, this is the first study describing the molecular adaptation of *M. graminicola* to host resistance.

## 2. Results

### 2.1. M. graminicola Transcriptome

In this study, we used *M. graminicola* RNA-Seq data retrieved from 14 (7 × 2 replicates) cDNA libraries sequenced in our previous works [10,22]. Libraries were obtained from pre-parasitic *M. graminicola* J2s, and from *O. sativa* cv Nipponbare (susceptible) or *O. glaberrima* TOG5681 (resistant) plants challenged with *M. graminicola*. Root tips and galls, when formed, were collected at 2, 4, or 8 dpi. Time points 2, 4, and 8 dpi were chosen to focus on the early stages of the interaction, when gene expression differences may determine the nature of interaction between both partners.

In our previous study, we generated a de novo *M. graminicola* transcriptome composed of 66,396 transcripts, obtained from the pre-parasitic J2s and the susceptible libraries reads [10]. Combined Blastx results indicated that only 52% of the *M. graminicola* sequences had similarities with recorded proteins from international databases [10], including with 14,875 (73%) of the 20,359 proteins predicted for *M. incognita* in a first genome version [26]. Since then, a draft genome was described for *M. graminicola*, with 10,895 predicted genes [24], and the *M. incognita* genome (third version) was much improved with 43,718 Coding DNA Sequences (CDS) [25]. Blast homology searches for the 66,396 assembled sequences, including now these 2 new genomes, were performed and we benefited from the results to clean our transcriptome. We eliminated sequences carrying the 2 following criteria: no hit in blast analyses and very low expression levels (Counts Per Million (CPM) values < 1 in all libraries). Moreover, we eliminated sequences with single expression data (reads detected in only one library) or two expression data when reads were not detected in replicate libraries, except sequences recording hits in the *M. graminicola* draft genome. Hence, we propose here a novel *M. graminicola* transcriptome version named Mg_v2, composed of 44,137 sequences (Table S1).

In detail, Blastn homology searches for the 44,137 Mg_v2 sequences against the 10,895 *M. graminicola* predicted CDS by Somvanchi et al. [24] retrieved 22,489 Mg_v2 sequences with at least one hit (51%) and against the genomic contigs retrieved 25,392 sequences (58%) (Table S1). In turn, 87% (9511) of the 10,895 predicted CDS [24] gave at least one hit on the 44,137 Mg_v2 sequences. This result indicates that the Mg_v2 transcriptome correctly covered the *M. graminicola* draft genome, considering that all the nematode stages were not sampled here. Blastx performed against the 43,718 *M. incognita* predicted proteins [25] found significant hits for 27,340 sequences (62%) of the Mg_v2 transcriptome, including 8111 sequences having no homologies with any of the *M. graminicola* genomic sequences produced by Somvanchi et al. [24], thus probably pointing to genes lacking in the *M. graminicola* draft genome sequence. Finally, taking into account of Blastx results against *Meloidogyne hapla* [27], *Caenorhabditis elegans* [28], and the Uniprot databases, we determined that the Mg_v2 transcriptome contains 7095 sequences (16%) with no homology at all.

As expected, the percentage of reads that mapped on the *M. graminicola* Mg_v2 transcriptome increased with time in the rice -nematode dual RNA-Seq libraries (Table S1). In the resistant libraries the nematode reads varied from 0.76–0.95% at 2 dpi to 3.81–4.34% at 8 dpi. In the susceptible RNA-Seq libraries, the percentage of *M. graminicola* mapped reads increased from 0.41–0.58% at 2 dpi to 4.63–9.70% at 8 dpi. The nematode reads number along time is consistent with microscopic observation data showing that in our inoculation conditions almost the same number of J2s were present in Nipponbare and TOG5681 roots after 7 days (Figure S1, [22]). However, all J2s in Nipponbare were feeding, had a sausage-like swollen body shape, when most of J2s were still vermiform in TOG5681 roots and could not reproduce further. Thus, the highest reads number in the susceptible RNA-Seq library at 8 dpi reflects a higher amount of nematode tissues in Nipponbare roots rather than a higher nematode number.

### 2.2. Differential Gene Expression Analysis from M. graminicola Transcriptomes in Susceptible and Resistant Rice Plants

Count files were generated from mapped reads to determine the nematode gene expression levels in each library. Using the EdgeR software, the expression of 36,121 *M. graminicola* transcripts could be examined across all samples (see M&M). Comparative gene expression analyses were performed to identify differentially expressed genes (DEGs) either up- or down-regulated between samples.

**Figure 1:**
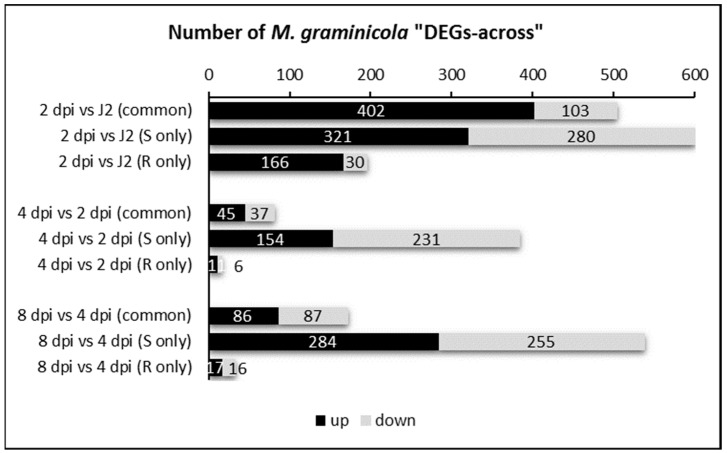
Number of *Meloidogyne graminicola* Differentially Expressed Genes across (“DEGs-across”) the early infection steps of rice susceptible (S) or resistant (R) plants.

The number of nematode DEGs across time was determined in susceptible (S) and resistant (R) samples by comparing the expression of genes at 2 dpi to that in pre-J2 stage, at 4 dpi to that at 2 dpi, and at 8 dpi to the expression at 4 dpi. In total, 2531 DEGs were identified, either common to R and S plant interaction or specific to one interaction (R only, S only) (Figure 1, Table S2). Overall, the highest number of nematode DEGs across was found between 2 dpi and the pre-J2 stage with more than 700 DEGs in R and 1100 DEGs in S, including 500 common to either R or S sample. Afterwards, much fewer nematode genes were regulated along time during the infection of resistant plants than during infection of susceptible plants (78% and 71% less DEGs at 4 and 8 dpi, respectively). This result may reflect that the majority of J2s in resistant plants did not suffer much change along this period, unable to feed and develop as well as they would in the susceptible plants.

**Figure 2:**
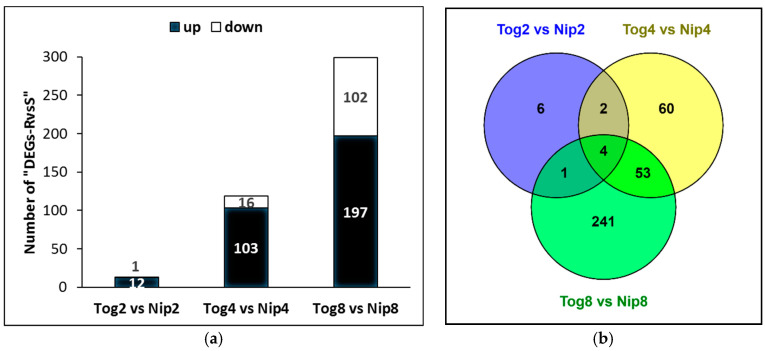
Number of *M. graminicola* Differentially Expressed Genes when comparing nematode infection of resistant (R) versus susceptible (S) rice plants (“DEG-RvsS”), at 2 dpi (Tog2 vs. Nip2), 4 dpi (Tog4 vs. Nip4) and 8 dpi (Tog8 vs. Nip8). (**a**) Number of “DEG-RvsS” at each time point. (**b**) Venn diagram showing specific and common “DEG-RvsS” at each time point.

The number of nematode DEGs between resistant versus susceptible plants (here named DEGs-RvsS) was determined by comparing the expression of genes in R versus S at each time-point (Figure 2, Table S3). In total, 431 DEGs were identified (312 up-regulated; 119 down-regulated) (Figure 2a), of which 367 were unique (Figure 2b). At 2 dpi, only 13 DEGs-RvsS were found (12 up- and 1 down-regulated in R compared to S). More differences in nematode gene expression occurred at 4 dpi with 119 DEGs-RvsS (103 up- and 16 down-regulated) and 8 dpi with 299 DEGs-RvsS (197 up- and 102 down-regulated). The majority of the DEGs-RvsS activated in R were already expressed by pre-J2s, but lowered or totally ceased their expression in the susceptible rice (Table S3). Conversely, all DEGs-RvsS activated in S were not expressed by pre-J2s, and not or poorly expressed in the resistant rice.

An annotation was associated with 67% of the DEGs-RvsS, which allowed identifying the functional categories impacted in *M. graminicola* metabolism or physiology. Among genes expressed without annotation (and with hit in the *M. graminicola* genome), two genes could be interesting: *Mg851* and *Mg1167* were both expressed in pre-J2s and later stages sampled; however, their expression was sustained at high level in resistant plants only (Table S3). Hereafter, we focus on the DEGs putatively involved in host infection and nematode development and highlight the differences we found in nematode gene expression during infection of resistant versus susceptible plants. As a matter of comparison, we also explored some categories highlighted in Shukla et al. [17].

### 2.3. Expression of Genes Related to Host Invasion

**Figure 3:**
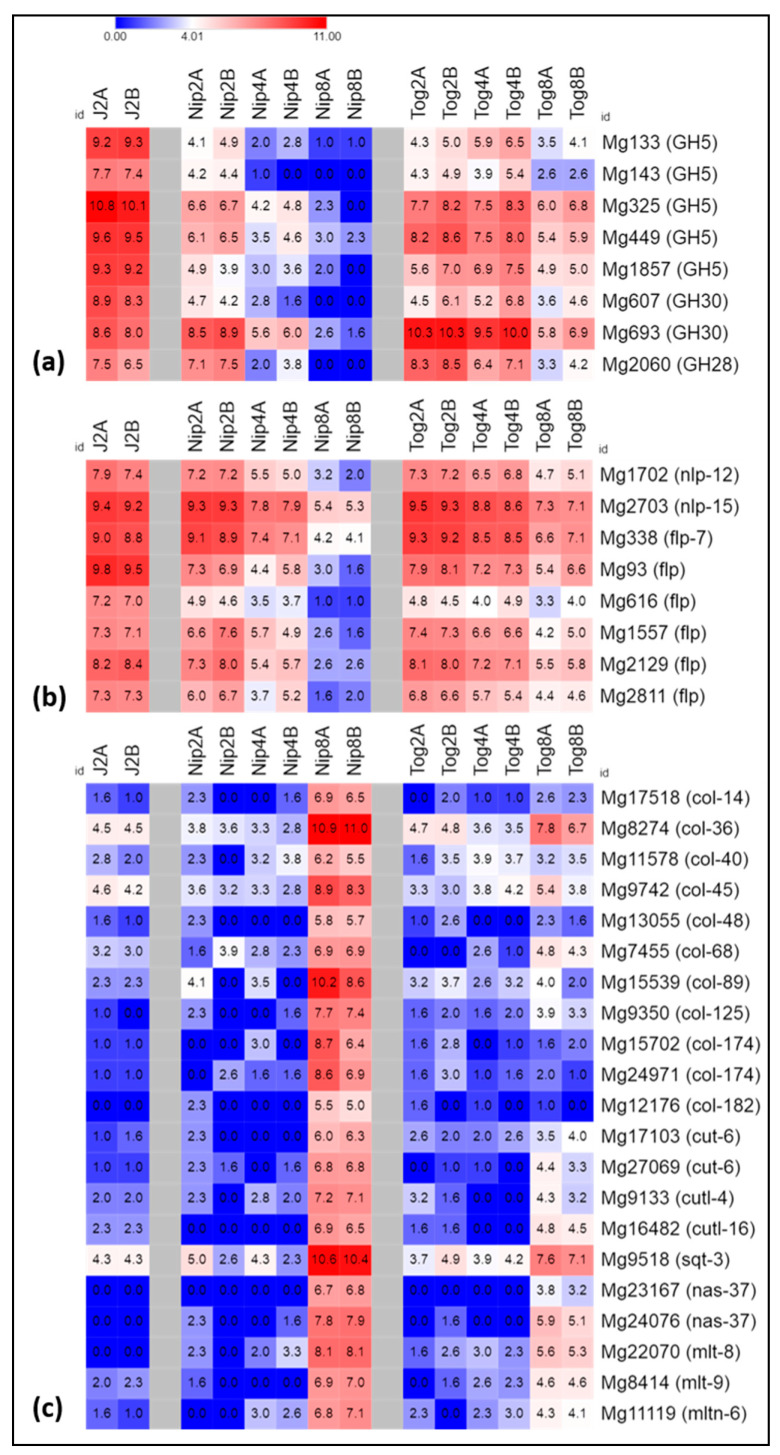
Schematic representation of the gene expression patterns of *Meloidogyne graminicola* differentially expressed genes (DEGs) encoding putative (**a**) cell wall-degrading enzymes (CWDE) (GH5: endoglucanase, GH30: glucuronoxylanase, GH28: polygalacturonase), (**b**) neuropeptides (*nlp*: neuropeptide like-proteins, *flp*: FMRFamide-relatided peptide-like), (**c**) molting-related proteins (*col*: cuticle collagen proteins, *cut*: cuticle, *cutl*: cuticlin-like, *nas*: zinc metalloproteinase, *mlt*: molting-defective, *mltn*: mlt10-related). Each row of the heatmap represents a gene and each column represents a stage of nematode development (J2: pre-parasitic J2s, Nip: in susceptible rice plants, Tog: in resistant rice plants) either at 2, 4 or 8 dpi. The common color key is given on the top of the figure. Values are expressed as Log2 of normalized Counts Per Million (CPM).

The *Meloidogyne* genome contains numerous genes encoding enzymes to facilitate root penetration and migration in host tissues, such as cell wall-degrading enzymes (CWDEs) or peptidases [26]. Twenty-one *M. graminicola* transcripts encoding predicted CWDEs able to degrade cellulose, hemicellulose and pectin polysaccharides, or encoding expansins, were consistently expressed (CPM >10 in several libraries) in the pre-J2s, R or S samples (Table S4). Eleven of the 15 genes expressed in pre-J2s remained also expressed during infection of resistant or susceptible plants at all time-points analyzed. However, among the 8 highly-expressed genes (CPM > 150) in pre-J2s, six were significantly down-regulated in susceptible plants from 4 dpi whereas their expression was sustained until 8 dpi in resistant plants, in particular, four endoglucanases (Cellulose-degrading enzyme, Glycoside Hydrolase family 5 (GH5)), one glucuronoxylanase (Hemicellulose-degrading enzyme *Mg693*, GH30) and one polygalacturonase (Pectin-degrading enzyme *Mg2060*, GH28) (Figure 3a, Table S4). In addition, an expansin gene (*Mg920*) exhibited the same expression pattern.

We also found 9 deregulated genes encoding peptidases, including cysteine, metallo, serine, and carboxy-peptidases. Most of them were either highly expressed in pre-J2s and/or at 2 dpi but their expression further dropped in susceptible plants but was sustained in resistant plants in the following time-points sampled (Table S4).

### 2.4. Expression of Genes Encoding Neuropeptides

Neuropeptides can modulate nematode behavior, including locomotion and chemo-attraction [29]. In *C. elegans*, 31 genes encode *flp* (FMRFamide-like peptides) and 75 genes encode *nlp* (neuropeptides-like proteins) [28]. The *M. incognita* neuropeptide complement is reduced compared to that of *C. elegans* with only 19 *flp* genes and 21 *nlp* genes readily identifiable [26]. A limited number (10) of *M. graminicola* genes encoding putative *nlp* or *flp* were identified in our transcriptomic dataset (Table S4). They shared the same expression patterns in resistant plants: they were overexpressed in pre-parasitic J2s and until 4 dpi, then their expression decreased at 8 dpi. However, as for CWDEs-encoding genes, *nlp* or *flp* genes expression lasted more in resistant plants than in susceptible plants and was significantly higher at 8 dpi (Figure 3b). Conversely, we noticed that genes encoding putative neuropeptide receptors (homologs of genes classified as “*npr*” in *C. elegans*) showed no significant alteration of their expression across the early stages of *M. graminicola* parasitism (Table S4).

### 2.5. Expression of Genes Related to Nematode Development and Metabolism

Root-knot nematodes undergo a series of molts once in the plant to reach the adult stage and reproduce. The first molt occurs after a determined feeding time (molt from J2 to J3) and is characterized by the acquisition of a second cuticle layer around larval body [30]. In the *M. graminicola* transcriptome, 85 genes encoding cuticle proteins (cuticle collagen proteins, cuticlins) or enzymes related to the molting process [31] were expressed. Among them, 21 *M. graminicola* genes were poorly activated during the infection of resistant plants compared to susceptible plants, where they were overexpressed at 8 dpi (Figure 3c, Table S4). When in J2s infecting susceptible plants, several developmental genes are clearly induced to evolve to J3 and ulterior stages, the same genes appear almost not induced in the J2s infecting resistant plants.

No change was apparent in genes involved in cytoskeleton (*unc*, *tubulin*, *actin*) except for one *actin-4* gene (*Mg2083*) which was activated at 8 dpi for nematodes infecting resistant plants (Table S3). Regarding genes involved in lipid metabolism, no change occurred with only one gene (*Mg8625*) encoding a fatty-acid hydroxylase which was more expressed at 8 dpi in nematodes infecting the susceptible host (Table S3).

### 2.6. Expression of Genes Related to Stress or Detoxification

*M. graminicola* genes putatively encoding enzymes related to detoxification of ROS or xenobiotic compounds, such as peroxiredoxins, catalases, superoxide dismutases, glutathione-S-transferases, glutathione peroxidases, were extracted from our expression dataset (Table S4). Some of these genes (*Mg1860*, *Mg2724*) were up-regulated after infection of resistant and susceptible plants (DEGs across) and only one gene (*Mg705*) sharing homology with a catalase, was considered a DEG-RvsS. *Mg705* expression was very high in pre-J2s and remained elevated in resistant plants until 8 dpi whereas it ceased to be expressed in susceptible plants from 4 dpi. These data indicate that detoxification genes are not required by the nematode during the infection of resistant plants.

### 2.7. M. graminicola Genes Encoding Putative Effectors

**Figure 4:**
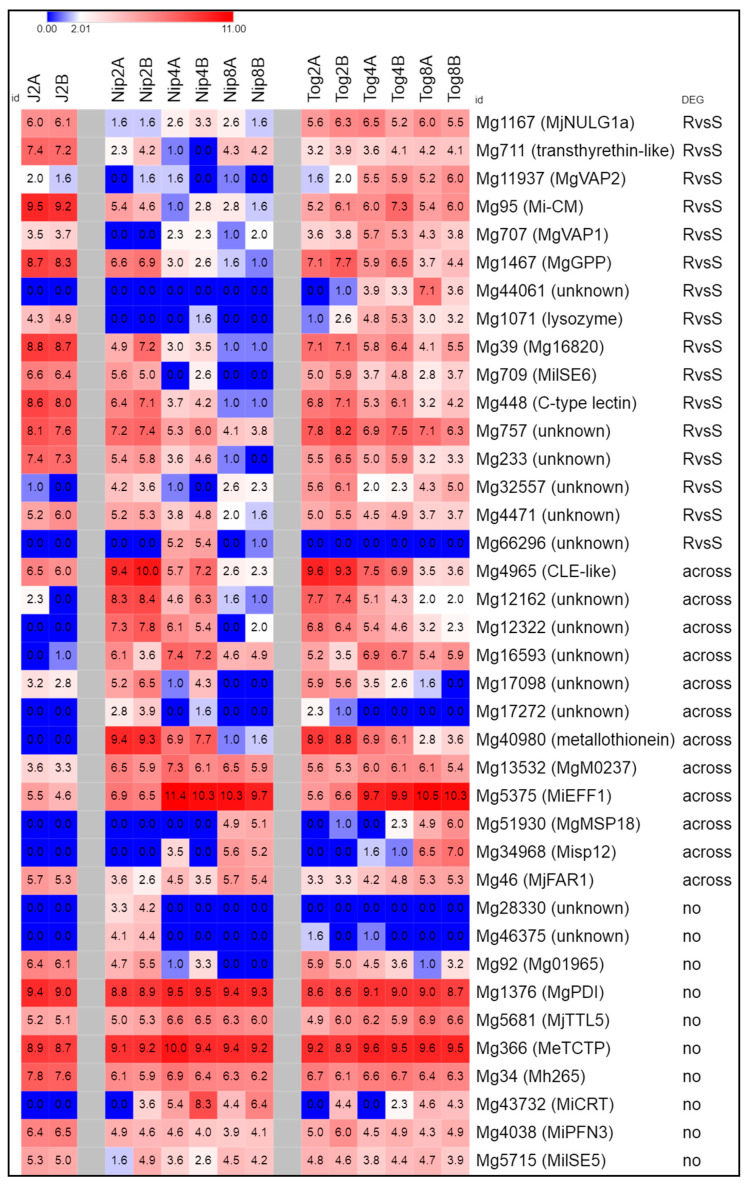
Schematic representation of the gene expression patterns of 38 *Meloidogyne graminicola* genes encoding candidate effectors or homologs of *Meloidogyne* effectors [10,11,12,13,14,15,32,33,34,35,36,37,38,39,40,41,42,43,44]. Each row represents a gene and each column represents a stage of nematode development (J2: pre-parasitic J2s, Nip: in susceptible rice plants, Tog: in resistant rice plants) either at 2, 4 or 8 dpi. The last column indicates if the gene was identified as a differentially expressed gene (DEG). The color key of the heatmap is given on the top of the figure. Values are expressed as Log2 of normalized Counts Per Million (CPM).

Among nematode molecules involved in host parasitism, several effector proteins others than CWDEs are secreted during infection. In the *M. graminicola* Mg_v2 transcriptome, 18 other transcripts encoding predicted secreted proteins (i.e., with a signal peptide and absence of a transmembrane domain) were evidenced as “DEGs-RvsS” (Tables S3 and S5). In addition, we searched for already known *M. graminicola* effectors and sequences homologs to *Meloidogyne* effectors that were lacking a detectable signal peptide in our dataset (Table S5). We verified that most of these genes were present in the *M. graminicola* draft genome even if they were not systematically identified as coding sequences (Table S5). Depending on effector gene, expression varied along time or stages, except *Mg1376* and *Mg366*, the closest *M. graminicola* orthologs of *MgPDI* [15] and *Me-TCTP* [32], respectively, which were highly expressed in all stages sampled (Figure 4, Table S5). The majority (78%) of the 38 genes surveyed are expressed by pre-J2s (Log2CPM > 1) and more than half (22 of 38) had similar expression pattern in the early stages of parasitism whatever the host genotype (Figure 4). The 16 other effectors or putatively secreted proteins surveyed were classified DEGs-RvsS.

Among *M. graminicola* effectors or candidate effectors, *Mg44061* (unknown function) was specifically expressed in resistant rice plants at 4 and 8 dpi. Conversely, *Mg66296* (unknown function) was not expressed at all in resistant plants, and only detected at 4 dpi in susceptible rice. *Mg707* and *Mg11937*, both encoding a venom allergen-like protein (VAP), were significantly up-regulated in resistant plants at 4 and 8 dpi. Expression of *Mg711* (transthyrethin-like), *Mg1467* (similar to *MgGPP* [11]), *Mg1071* (lysozyme), *Mg39* (similar to *Mg16820* [13]), *Mg448* (C-type lectin), *Mg757*, *Mg233*, *Mg32557* and *Mg4471* (unknown functions) were similar in pre-J2s and 2 dpi and decreased later, but to a lesser extent in resistant plants compared to susceptible plants.

Regarding the putative homologs of 12 virulence effectors characterized in other *Meloidogyne* species, the majority shared the same expression patterns during infection of resistant or susceptible rice plants (“DEG-across” or no alteration of their expression), except *Mg1167* (homology with *Mj-NULG1a*, [33]) which was down-regulated in susceptible plants, *Mg95* (homology with *Mi-CM,* [34]) and *Mg709* (homology with *MilSE6*, [35]) that were down-regulated in resistant plants later than in susceptible plants.

In addition to *Mg92* and *Mg448*, encoding proteins with a C-type lectin domain, we identified 6 other genes encoding proteins with a C-type lectin domain and a signal peptide for secretion which were highly expressed in pre-J2s and also down-regulated in resistant plants later than in susceptible plants (Table S3).

### 2.8. Validation of a Set of DEGs by RT-qPCR

Among the different categories of DEGs, we selected 11 *M. graminicola* genes encoding either CWDE (*Mg449* and *Mg693*), neuropeptides (*Mg1702* and *Mg2129*), VAP (*Mg707* and *Mg11937*) or candidate effectors (*Mg757*, *Mg4965*, *Mg12322*, *Mg13532*, and *Mg44061*) to validate their expression in RT-qPCR assays. We successfully cloned the cDNA sequences of *Mg693*, *Mg1702*, *Mg2129, Mg707* and *Mg44061* in this study, the others had been already cloned in Petitot et al. [10]. Specific primers were designed for each gene and used in RT-qPCR assays on RNAs extracted from pre-parasitic J2 and from susceptible or resistant rice plants after a 2-, 4-, or 8-day challenge with *M. graminicola*. Expression profiles were obtained for the 12 genes on 3 independent biological replicates (Figure S2). The ratio of nematode genes expression between resistant and susceptible plants gave values highly similar in qPCR and RNA-Seq data (Table 1).

### 2.9. Two Up-Regulated Genes in Rice Resistant Plants Encode Putative VAP Proteins

**Figure 5:**
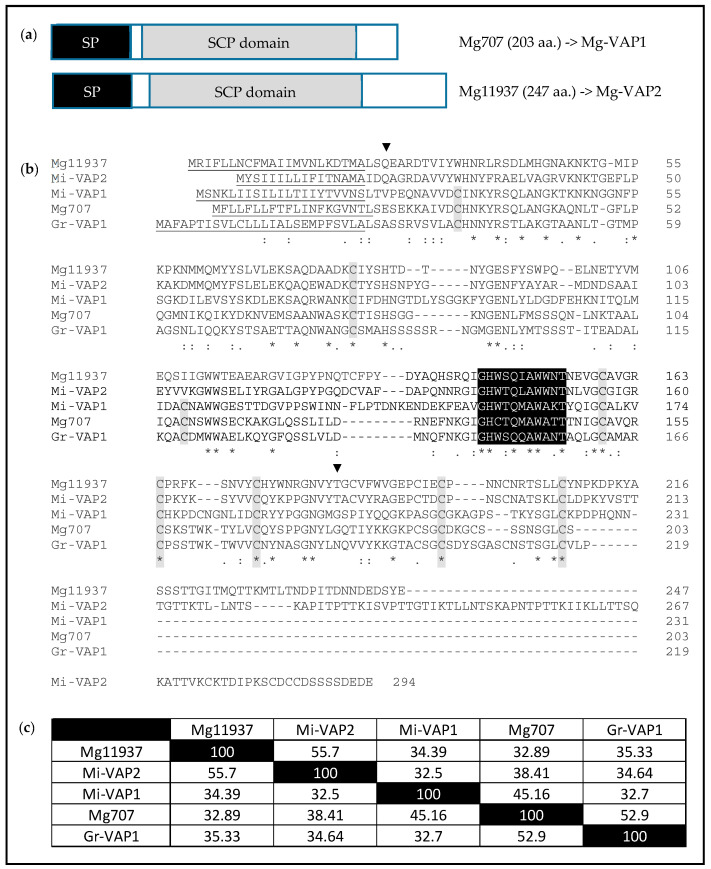
*Mg707* and *Mg11937* encode venom allergen-like proteins (VAPs). (**a**) Schematic representation of the two predicted proteins. (**b**) Protein sequences alignment of Mg707 and Mg11937 with *M. incognita* Mi-VAP1 (Genbank AAD01511, [47]), Mi-VAP2 (Genbank ABO38019, [48]) and *G. rostochiensis Gr-VAP1* (Genbank AEL16453, [46]): the signal peptides are underlined, the conserved cysteines are highlighted in grey, the Venom Allergen conserved site is highlighted in black and the SCP domain (annotated as Smart00198 in NCBI) is delimited by black triangles. (**c**) Percent identity matrix of the SCP domain sequences.

*Mg707* and *Mg11937* share high homologies with the *Gr-VAP1* gene from *Globodera rostochiensis*, an important effector able to modulate basal plant immunity [45,46]. *Mg707* and *Mg11937* putatively encode proteins of 203 and 247 aa., respectively, with a signal peptide and a Sperm-Coating Protein (SCP) domain characteristic of VAP proteins (Figure 5a), designed also as the cysteine-rich secretory proteins/antigen5/pathogenesis-related1 (CAP) domain. *Mg707* and *Mg11937* only share ~33% aa. identity. Deduced amino-acid sequences alignment of *Mg707* and *Mg11937* with *Mi-VAP1* [47], *Mi-VAP2* [48] and *Gr-VAP1* [46] indicated that *Mg707* is close to *Mi-VAP1* and *Gr-VAP1,* and *Mg11937* is close to *Mi-VAP2* (Figure 5b,c). Hence, we propose here to rename *Mg707* as *Mg-VAP1* and *Mg11937* as *Mg-VAP2*.

**Figure 6:**
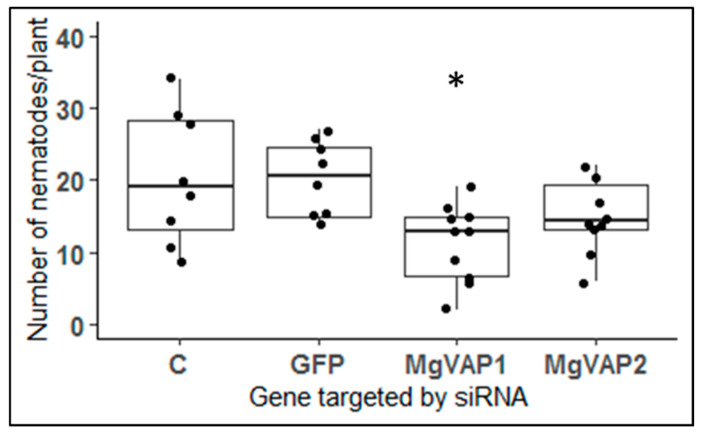
Effect of *Mg-VAP* genes silencing on nematode colonization. Number of nematodes observed in susceptible Nipponbare rice roots 8 days after plant inoculation with 100 J2s soaked in siRNAs targeting either *Mg-VAP1* or *Mg-VAP2*. As control treatments, nematodes were soaked in water (C) or in siRNAs targeting GFP. Boxplots represent data of one experiment (*n* ≥ 8 per treatment). Data were statistically tested for significance with ANOVA with post-hoc Tukey HSD (* *p* < 0.05). The experiment was repeated twice with the same results.

In order to study the role of *Mg-VAP1* and *Mg-VAP2* in *M. graminicola* parasitism, we performed in vitro gene silencing by soaking J2s with specific siRNAs. Silencing of the *Mg-VAP1* gene caused a significant reduction (*p* < 0.05) of nematode colonization in Nipponbare rice roots as compared to the control treatments (Figure 6). A small difference in the number of nematodes in rice roots was also observed for *Mg-VAP2* silencing but was not reported statistically significant from control treatments.

## 3. Discussion

Molecular mechanisms associated to resistance in plant-RKN interactions are poorly described and understood. Deciphering nematode gene expression patterns during the infection of resistant plants could provide insights into the RKN pathogenicity mechanisms. In this study, we compared the *M. graminicola* transcriptome in resistant and susceptible rice plants during 8 days after inoculation to examine the nematode behavior in a resistant host and identify novel *M. graminicola* genes involved in host parasitism.

### 3.1. Desperately Seeking to Settle

Of the 36,121 *M. graminicola* transcripts surveyed in this study, only 367 genes were differentially expressed between resistant and susceptible plants datasets (DEGs-RvsS). Globally, we did not observe expression of specific nematode genes but rather a different dynamic of gene expression associated to nematode infection in the resistant rice genotype. For instance, whether entering resistant or susceptible host roots, the nematode (pre-J2s) produce a large set of degrading enzymes, including CWDEs, peptidases as well as a series of other proteinaceous effectors (Figure 3). However, expression of the enzyme-coding genes sharply decreases in the susceptible host after 2 days when *M. graminicola* initiate their feeding sites in the vascular cylinder. On the contrary, expression of the same genes in the resistant host lasts longer and is still sustained after 8 days when only a limited number of feeding sites are initiated. The same expression pattern applies for nematode genes encoding neuropeptides, some might be involved in chemotaxis and movement. Conversely, RKN genes involved in development are poorly expressed when the nematodes try to infect the resistant host but are activated after 4 to 8 days in the susceptible plants where the nematodes successfully settle and start to develop into J3 (Figure 3). Escaping these general trends in expression patterns are several effector genes that are expressed at different time points (Figure 4) and a few genes with unknown function that might deserve interest (Table S3).

To our knowledge, only two studies finely described the RKN genes regulation during the interaction with resistant hosts, namely in the resistant African horned melon *Cucumis metuliferis* [16] and in tomato [17]. Ling et al. [16] focused on the secretome of *M. incognita* in *C. metuliferis* when Shukla et al. [17] performed a comprehensive comparative analysis of gene expression of *M. incognita* infecting either susceptible tomato (*Solanum lycopersicum*) lines or a resistant transgenic tomato line expressing the *Mi* resistance gene. Our observations of RKN gene regulation in resistant rice are similar to the conclusions of Shukla et al. [17], who indicated over-expression of *M. incognita* genes encoding glucosyl hydrolases, pectate lyases, and peptidases in the resistant tomato as opposed to RKN genes involved in host parasitism, development and defence in the susceptible host. Thus, a global view of the nematode behavior emerges, indicating that while J2s settle in susceptible plants and cease the production of CWDE, peptidases and neuropeptides transcripts, the nematodes still require synthesizing such compounds in resistant plants.

To facilitate root penetration and migration in host tissues, plant-parasitic nematodes produce cell-wall modifying enzymes, including CWDEs able to degrade cellulose, hemicellulose and pectin polysaccharides and other enzymes to soften the cell-wall structure (see [7] for review). These genes were likely inherited through lateral genetic transfer from soil bacteria or fungi [49] enabling nematodes to successfully invade plant tissues. For sedentary nematodes, expression of CWDE genes generally ends once the nematodes settle and enter in the sedentary phase [7]. In rice- (this study) and tomato-resistant [17] plants, gene expression is sustained, suggesting that the nematode have more difficulties to migrate in the resistant roots and/or to settle to establish a feeding site.

Conversely, after initiation of a permanent feeding site, parasitic J2 nematodes feed and develop to J3, J4 and adults through three successive molts during nematode ontogenesis [30]. In susceptible plants, numerous *M. graminicola* genes related to molting process are induced at 8 dpi, including those encoding cuticle collagen proteins, cuticlin proteins, or enzymes involved in molting and the shedding of the old cuticle [10]. In rice- (this study) and tomato-resistant [17] plants, these genes are slightly induced or not induced at all. These molecular data are in accordance to histological observations which showed that, despite the fact that nematodes are able to penetrate roots, almost no parasitic J2s are able to evolve to further developmental stages in resistant rice plants [22] or in *Mi*-expressing tomato plants [17]. Similar data were obtained for the root lesion nematode *Pratylenchus penetrans* during infection of alfafa (*Medicago sativa* L.) where several transcripts encoding collagen proteins of the nematode cuticle were among the genes highly expressed in the susceptible cultivar but not in the resistant cultivar [50].

### 3.2. Facing Host Defenses

Plant-parasitic nematodes produce a range of antioxidant proteins to evade plant defense responses during infection [7,51]. We found only one catalase gene (*Mg705*) highly expressed in J2s and until 8 dpi in resistant rice compared to susceptible plants where expression ceases after 2 or 4 dpi. The *M. incognita* homolog of the catalase gene (*Minc10184*–*Minc3s00005g00340*) was also up-regulated during the resistance response of *Mi*-expressing tomato [17]. It is likely that they play a role in detoxification by protecting the nematode cells from oxidative damage by ROS, namely H_2_O_2_ that may be produced during root invasion. However, it seems that *M. graminicola* did not display a stronger detoxification strategy when infecting resistant plants as compared to susceptible plants. In the resistant rice TOG5681-*M. graminicola* interaction, resistance expresses at several infection stages, reducing J2s penetration, giant cell expansion and further female development, and HR-like cell death was not observed [22]. The level of ROS production in response to nematode attack may be similar in resistant and susceptible rice plants and may not fully explain the resistance of TOG5681. In contrast, Shukla et al. [17] highlighted the expression of several RKN genes involved in cytoskeleton and starvation stress-induced apoptosis in addition to detoxifying enzymes in the resistant tomato host. Expression of the *Mi* resistance gene in tomato induces localized cell death when J2s attempt to establish a feeding site [52] and might therefore kill some larvae inside roots. Here, we did not observe specific changes in expression of cytoskeleton and starvation-related genes in *M. graminicola* when infecting the resistant rice, which is consistent with an absence of HR-like response. It will be interesting to investigate whether similar expression trends would be conserved in other rice (*O. sativa*) genotypes displaying partial or full *M. graminicola* resistance recently described [18,19,20].

Transcriptomic analyses showed that rice genes predicted to be involved in defence responses, phenylpropanoid and hormone pathways were strongly induced from 2 dpi and later on in TOG5681, in contrast to the susceptible plants where these genes were induced later and less strongly [22]. At 2 dpi, we detected only minor differences in the transcriptomes of nematodes infecting resistant or susceptible rice plants but major changes occurred at 4 dpi (119 DEGs-RvsS) and 8 dpi (299 DEGs-RvsS) indicating an effect of the plant genotype on the nematode gene regulation. In fact, the majority of the DEGs-RvsS activated in the resistant rice were genes already expressed by pre-J2s that lowered or totally ceased their expression in the susceptible rice. These genes were almost all involved in the plant invasion process (cell wall-degrading or -modifying enzymes, neuropeptides), and detoxification or evasion of plant defense responses. Thus, our data suggest that these genes are required at an early RKN stage for infecting the plant, irrespective of their level of resistance to the nematode.

By taking into account the transcriptomic analysis of pre-J2s, our study also provides with valuable understanding of changes that appear in the nematodes in the very first days after infecting a resistant or susceptible host plant, rather than merely highlighting changes occurring between contrasted host genotypes. For instance, Shukla et al. [17] discussed up-regulation of *M. incognita* genes related to cell-wall degradation and peptidases in the resistant tomato cultivar. In the same way, Vieira et al. [50] stated that nematode genes critical for *P. penetrans* development and encoding collagen proteins were down-regulated during infection of the resistant alfalfa cultivar. However, knowledge of gene expression in the pre-infective stage would be required to verify if the same pattern occurred for *M. incognita*, *P. penetrans* and *M. graminicola*.

### 3.3. M. graminicola Express Their Core Set of Effectors

Our comparative transcriptomic data also showed that *M. graminicola* relies on the secretion of a set of effector proteins, in addition to CWDEs or peptidases, to establish infection within rice, whether susceptible or resistant cultivars. Effectors are produced by pathogens to facilitate parasitism and a small amount of *M. graminicola* secreted proteins have been described [11,12,13,14,15]. Mining the *M. graminicola* transcriptome returned eight novel candidate effectors in addition to those previously described in Petitot et al. [10]. Furthermore, we identified the orthologs of 12 effectors characterized in other *Meloidogyne* species (Table S5).

Indeed, we found that the majority of the 38 effector or candidate effector genes surveyed are expressed by pre-J2s (Figure 4). We showed that half of them have similar expression pattern in the early stages of parasitism and can be actively transcribed, whatever the host genotype. For instance, *Mg1376* and *Mg366*, the closest *M. graminicola* orthologs of *MgPDI* [15] and *Me-TCTP* [32], respectively, were highly expressed at all times sampled in both rice genotypes, which is consistent with their probable role in parasitism. *MgPDI* and *Me-TCTP* are expressed in pre-J2s esophageal glands and activated during plant infection until late developmental stages [15,32]. *MgPDI* encodes a disulfide isomerase probably secreted in the apoplast where it may be required for protecting the nematode from oxidative damage [15]. *Me-TCTP* belongs to the translationally controlled tumor protein (TCTP) family and might be delivered by the nematode to the host cell cytoplasm where it is able to suppress programmed cell death and, by extension, plant immune defense responses [32]. Thus, expression of these two genes by *M. graminicola* are probably required for counteracting some host defense reactions and protecting the nematode.

In contrast, 16 DEGs-RvsS were identified among the 38 effectors and putatively secreted proteins surveyed (Figure 4). Like for CWDEs or peptidases, the majority of these DEGs-RvsS were expressed in pre-J2s and down-regulated in resistant plants later than in susceptible plants. This trend included several candidate effectors identified here or previously [10], *MgGPP* [11], *Mg16820* [13] and the closest orthologs of *Mj-NULG1a* [33], *MilSE6* [35], and the chorismate mutase *Mi-CM* [34]. Like *MgPDI* and *Me-TCTP*, *MgGPP* is another effector that may be involved in the suppression of defense reactions of the host cell to promote parasitism [11]. Among the *M. graminicola* genes highly expressed in pre-J2s, *Mg448* encodes a C-type lectin that could also be involved in overcoming the host defenses as reported for *Mg01965* [14] and for the cyst nematode, *Heterodera glycines* [53].

We identified here two other DEGs-RvsS whose expression profile deserved attention: *Mg44061* was specifically expressed in resistant rice plants, only. Conversely, *Mg66296* was not expressed at all in resistant plants and was specifically expressed at 4 dpi in the susceptible plants. No homology could be found for *Mg44061* and *Mg66296* in databases and both putatively encoded proteins have a predicted signal peptide for secretion. Functional analyses are required to assess the importance of these novel *M. graminicola* pioneer genes for parasitism.

### 3.4. A Role for VAP Genes during Infection of Resistant Plants?

*Mg-VAP1* and *Mg-VAP2* are specifically induced when *M. graminicola* infects resistant rice TOG5681 and silencing of the *Mg-VAP1* gene affects *M. graminicola* parasitism. VAPs belong to a structurally conserved group of secreted proteins abundantly secreted during several stages of parasitism by plant- and animal-parasitic nematodes [54]. Their name originated from the allergenic protein “antigen 5” identified in the white-faced hornet venom [55]. Importantly, VAPs are key players in the modulation of plant immunity. Knocking down the expression of *VAP* genes can severely affect nematode virulence [45,56,57,58]. In particular, expression of *VAP* genes are required for the onset of parasitism at the time of root invasion and migration through host tissues [58,59]. Conversely, ectopic expression of *G. rostochiensis* or *Heterodera schachtii VAP* genes in potato or *Arabidopsis* significantly increases plant susceptibility to nematode infection [46]. In addition, *A. thaliana* lines overexpressing nematode *VAP* genes loose basal immunity to unrelated pathogens, including fungi, bacteria, and oomycetes [46]. Modulation of plant immunity involves interaction of nematode VAPs with host proteases [45,57]. In tomato, *Gr-VAP1* specifically interacts with and inhibits the apoplastic papain-like cysteine protease (PLPC) Rcr3pim, increasing the susceptibility of tomato plants to *G. rostochiensis* [45]. Additionally, Luo et al. [57] showed that ectopic expression in tobacco cells of the protein HaVAP2 (minus its signal peptide) from the cereal cyst nematode *H. avenae* allowed its transfer into the host cell nucleus where it can interact with the *Hordeum vulgare* HvCLP peptidase. VAPs also contain a SCP/CAP domain able to bind lipids, such as leukotrienes and sterols, but little is known about endogenous ligands that are bound during parasitism [54]. Regarding the importance of VAPs in the first steps of plant-nematode interactions, especially through the modulation of plant immunity, one can easily imagine that *Mg-VAP1* and *Mg-VAP2* have an important role to play in *M. graminicola* parasitism. Accordingly, we showed that silencing the *Mg-VAP1* gene had a negative effect on the nematode ability to colonize rice roots. However, we did not observe any significant effect of *Mg-VAP* genes in cell death suppression assays conducted in tobacco (data not shown) as observed for some *VAP* genes [46]. Up-regulation of *Mg-VAP1* and *Mg-VAP2* genes when the nematode infects resistant rice plants may reflect a special requirement of their function to counteract host immunity, even if it is finally not enough to overcome rice resistance. What their role is and what their targets are in rice-compatible interactions remain to be elucidated.

To conclude, this study gives for the first time a global view of *M. graminicola* genes expressed in the first steps of interaction with resistant rice plants and provides with novel nematode gene sequences. This work highlighted that *M. graminicola* adapts its gene expression depending on the plant genotype. In a hostile host environment, the nematode still (desperately) tries to infect the plant as in a susceptible host, but may also express specific genes, including those encoding *VAPs*. It will be interesting to investigate how these genes help the nematode to cope with the plant resistance responses and whether they could participate to break rice resistance to *M. graminicola*.

## 4. Materials and Methods

### 4.1. Nematode Population

The population of *M. graminicola* was originally collected from Laurel (Batangas, Philippines) and cultured on *O. sativa* cv. IR64 in a growth chamber as described in Petitot et al. [10].

### 4.2. Nematode Inoculation Assays on Rice Plants

Seeds of *O. sativa* (cv. Nipponbare, susceptible) or *O. glaberrima* (TOG5681 accession, resistant) were germinated on sand wetted with Hoagland ¼ solution (KNO_3_ 5 mM; KH_2_PO_4_ 1 mM; Ca(NO_3_)_2_ 5 mM; MgSO_4_ 2 mM; 25 mg iron; trace element) for 7 days and then transferred to plastic tubes containing 10 g Sand and Absorbent Polymer (SAP) substrate [60] wetted with Hoagland ¼ solution. Rice plants were maintained in a growth chamber under controlled conditions at 26 °C/24 °C day/night temperature, under a 14 h/10 h day/night light regime and 70% relative humidity. Three days after transplanting into SAP substrate, plantlets were inoculated with freshly hatched J2s in 1 mL of demineralized water. We used 100 J2s to inoculate the Nipponbare plantlets and 400 J2s for the TOG5681 plantlets, which allowed a similar level of root colonization by the nematode during the 8-day time course analysis without modifying the final outcome of the interaction in Nipponbare (susceptible) and TOG5681 (resistant) (Figure S1, [22]). One day after inoculation, plants were transferred to a 50 mL hydroponic culture system containing Hoagland 1/4 solution, in order to stop J2 penetration and synchronize the infection process between plants. Roots tips (1–2 mm) or visible galls were collected at 2, 4 and 8 dpi and pooled from 25 plants at each time point. Samples were immediately frozen in liquid nitrogen and stored at −80 °C until further use. Three independent biological replicates were performed, 2 of them were used for RNA-Seq and the 3 replicates were used for qPCR validation assays.

### 4.3. cDNA Libraries and RNA-Seq Data

We used RNA-Seq data from 14 cDNA libraries produced in our previous works (ENA/SRA accession number ERS715982, [10,22]). Two replicate libraries (J2A and J2B) were obtained from freshly hatched pre-parasitic *M. graminicola* J2s, and six (3 × 2 replicates) libraries were obtained from *M. graminicola*-infected *O. sativa* cv Nipponbare (susceptible) root tips and galls collected at 2 dpi (Nip2A and Nip2B), 4 dpi (Nip4A and Nip4B), or 8 dpi (Nip8A and Nip8B) [10]. Six (3 × 2 replicates) libraries came from *M. graminicola*-infected *O. glaberrima* TOG5681 (resistant) root tips or galls, when formed, also collected at 2 dpi (Tog2A and Tog2B), 4 dpi (Tog4A and Tog4B) and 8 dpi (Tog8A and Tog8B) [22].

### 4.4. Functional Annotation of Genes

Using the 66,396 *M. graminicola* transcripts described in Petitot et al. [10], Blastn and Blastx alignments were performed against 3 datasets: the 10,895 predicted genes and the 4304 contigs from the draft genome of *M. graminicola* (Bioproject PRJNA411966, [24]) and the 43,718 CDS of the *M. incognita* protein database (Bioproject PRJEB8714, [25]) deposited at the WormBase ParaSite website [61]. In addition, Blastx alignments were performed on the *M. hapla* and *C. elegans* protein databases and the UniProt database, and PFAM and Interproscan domains were determined for *M. graminicola* predicted proteins [10]. To retrieve putative homologs of functional genes, PFAM and Interproscan accession numbers, *C. elegans* gene class names, as described in WormBase [28], or keywords were used.

For the identification of putative homologs of effector genes identified in *M. graminicola* or other *Meloidogyne* species, tblastn were performed with protein sequences against the *M. graminicola* transcript sequences. An e-value threshold of 1 × 10^−10^ was applied and the best hit was kept.

To improve the *M. graminicola* secretome established in Petitot et al. [10], we recovered read pairs that did not map on the *O. sativa* Nipponbare genome (MSU7), the *O. glaberrima* TOG5681 genome [62] and the *M. graminicola* transcriptome v1. We performed a de novo assembly of the *M. graminicola* transcriptome including these novel reads. However, no new candidate effector was identified when performing further bioinformatics analyses described in Petitot et al. [10].

### 4.5. Differential Expression Analyses

Reads from all the libraries were mapped to the *M. graminicola* transcriptome with the BWA-ALN software [63]. BAM files were converted to count files using Samtools idxstat. Normalization and differential gene expression analyses were performed on all the count files using EdgeR v3.24.1 [64,65] installed on the Galaxy instance hosted by the IRD bioinformatics computing cluster (http://itrop-galaxy.ird.fr). Differential expression of one considered transcript was analyzed only if at least two samples had a CPM value > 1. Genes were considered as induced or repressed only when their log2FC was >2 or <−2, respectively, and their False Discovery Rate (FDR) was <0.05 and designed as DEGs. Data lists were compared with the Venny 2.1 tool [66]. The heatmaps were constructed with the Morpheus software (https://software.broadinstitute.org/morpheus).

### 4.6. Cloning of M. graminicola Candidate Genes and Sequences Analyses

Some *M. graminicola* genes were already cloned in Petitot et al. [10] (Genbank accession numbers MF166620-166634). For novel selected genes (*Mg693*, *Mg1702*, *Mg2129*, *Mg707* and *Mg44061*), primers were designed from the assembled sequences (Table S6) and synthesized (Eurogentec, France). cDNAs were cloned as described in Petitot et al. [10] and Sanger sequenced (Genewiz, Germany) (Genbank accession numbers MT349880–349885). The presence of signal peptide was predicted using the SignalP 5.0 server [67]. The Clustal-Omega alignment tool [68] was used to align the VAP protein sequences and establish the percent identity matrix.

### 4.7. Validation of DEGs by RT-qPCR

Specific primers were designed from the *M. graminicola* cloned sequences using the Beacon Designer 7.0 software (Premier Biosoft International, Palo Alto, CA, USA) (Table S6). RT-qPCR assays were conducted on RNAs extracted from pre-parasitic J2 and from susceptible or resistant rice plants after a 2-, 4-, or 8-day challenge with *M. graminicola*. We used the duplicate RNAs samples from the sequenced libraries and produced a third replicate in this study. The qPCR assays were performed on cDNA samples (diluted 1: 100) as described previously [10]. Amplifications were carried out in an Mx30005P thermal cycler (Stratagene, USA) using the Takyon Kit for SYBR assays (Eurogentec, France). The SATQPCR tool (http://satqpcr.sophia.inra.fr/cgi/home.cgi) was used to calculate the relative expression between the samples R and S at each time-point using the 2^−ΔΔCt^ method. The *M. graminicola* actin gene and *Mg17272* were chosen by the SATQPCR tool as reference genes to normalize data. Statistical analysis of RT-qPCR data was performed to test whether the means were different (Student t-test; statistical significance *p* < 0.05).

### 4.8. In-Vitro Silencing of Mg-VAP Genes

The design and construction of siRNAs were performed according to the Silencer^®^ siRNA Construction Kit (Life Technologies, Carlsbad, CA, USA) (Table S6). The siRNA-mediated silencing of *Mg-VAP* genes was performed according to Arguel et al. [69]. Briefly, 10,000 freshly hatched *M. graminicola* J2s were soaked in 40 µL mineral water (Volvic, Volvic, France) containing 50 ng/µL siRNAs for one hour. As control treatments, nematodes were soaked in water or in 50 ng/µL siRNAs targeting GFP [9]. Nematodes were washed twice and suspended in 100 µL of water for 16 h. One-hundred soaked-J2s were used for inoculation of Nipponbare plants as described above. Eight days after inoculation, root systems were collected and incubated with acid fuchsin to stain nematodes [70] and counted. To analyze significant differences between the control and treatment groups, statistical analysis was performed with R [71] for one-way analysis of variance (ANOVA) with a post hoc Tukey honest significant difference (HSD) test for multiple comparisons. Data were considered significant when *p*-value < 0.05.

## Figures and Tables

**Table 1:** Validation of RNA-Seq data by quantitative PCR (qPCR). Values are expressed as Log2-Fold Change. For RNA-Seq, two independent biological assays were analysed (replicates A and B) and * indicate DEGs as defined by EdgeR (log2FC > 2 or <−2, padj < 0.05). For Q-PCR, three independent biological assays were analysed (replicates A and B, both used for RNA-Seq, and replicate C) and * indicate significant differences (*t*-test, *p* < 0.05) between resistant (R) and susceptible plants (S).

*M. graminicola* Transcript	Predicted Function	RNA Seq (R versus S)			qPCR (R versus S)		
		2 dpi	4 dpi	8 dpi	2 dpi	4 dpi	8 dpi
*Mg449*	Endoglucanase	2.17 *	3.69 *	2.99 *	2.70 *	5.14 *	5.45 *
*Mg693*	Glucuronoxylanase	1.57	3.95 *	4.38 *	1.88	3.66 *	5.82 *
*Mg1702*	Neuropeptide “*nlp*”	0.07	1.40	2.19 *	0.70	2.40 *	4.98 *
*Mg2129*	Neuropeptide “*flp*”	0.33	1.58	3.14 *	1.03	2.06	4.69 *
*Mg707*	VAP	9.26	3.27 *	2.57 *	2.42	4.99 *	5.62 *
*Mg11937*	VAP	1.38	5.03 *	5.86 *	2.08	5.63 *	6.78 *
*Mg757*	Unknown	0.67	1.57	2.8 *	1.11	2.83 *	4.40 *
*Mg4965*	CLE-like	−0.26	0.60	1.11	1.09	0.96	1.19
*Mg12322*	Unknown	−0.92	−0.72	1.68	−0.81	−0.15	3.67
*Mg13532*	Unknown	−0.79	−0.75	−0.44	−0.49	−0.27	1.04
*Mg44061*	Unknown	5.42	9.55	12.09 *	0.001	1.89	5.02

## Supplementary Materials

The following are available online at https://www.mdpi.com/2076-0817/9/8/644/s1, Figure S1: *Meloidogyne graminicola* colonization and reproduction in resistant TOG5681 (R) and susceptible Nipponbare (S) rice roots, Figure S2: Expression profiles of *M. graminicola* genes determined by quantitative RT-PCR experiments, Table S1: Summary statistics for the *M. graminicola* Mg_v2 transcriptome, Table S2: List of *M.graminicola* “DEGs-across” the early infection steps of rice susceptible or resistant plants, Table S3: List of *M. graminicola* “DEGs RvsS” when comparing nematode infection of resistant plants versus susceptible plants, Table S4: List of *M. graminicola* gene families and their expression in rice susceptible or resistant plants, Table S5: List of *M. graminicola* effector or candidate effector genes, their annotation and their expression in rice susceptible or resistant plants, Table S6: Sequences of primers used for gene cloning, qPCR and soaking.
