# Supplementary material for: Analyses of the Root-Knot Nematode (Meloidogyne graminicola) Transcriptome during Host Infection Highlight Specific Gene Expression Profiling in Resistant Rice Plants
Source: Pathogens. 2020 Aug 8;9(8):644. doi: 10.3390/pathogens9080644 (PMC7460394; doi:10.3390/pathogens9080644)

Figure S1. *Meloidogyne graminicola* colonization and reproduction in resistant TOG5681 (R) and susceptible Nipponbare (S) rice roots. Nematode colonization and reproduction were determined after plant inoculation with 100 (for S) or 400 (for R) second-stage juveniles (J2). **A.** Number of nematodes stained with fuchsin in R and S rice roots at 7 days after plant inoculation. Boxplots represent data of three independent biological replicates with 10 plants per condition (n =5;3). Mean nematode numbers were not statistically different with Kruskal-Wallis rank sum test (Kruskal-Wallis chi-squared = 13.253, df = 8, p-value = 0.1034). **B**. Number of next-generation J2 obtained from R and S rice roots collected at 35 days after plant inoculation. Boxplots represent data of three independent biological replicates with 16 plants per condition (n =8;3). Mean nematode numbers were statistically different with Kruskal-Wallis rank sum test (Kruskal-Wallis chi-squared = 41.913, df = 5, p-value = 6.135e-08).

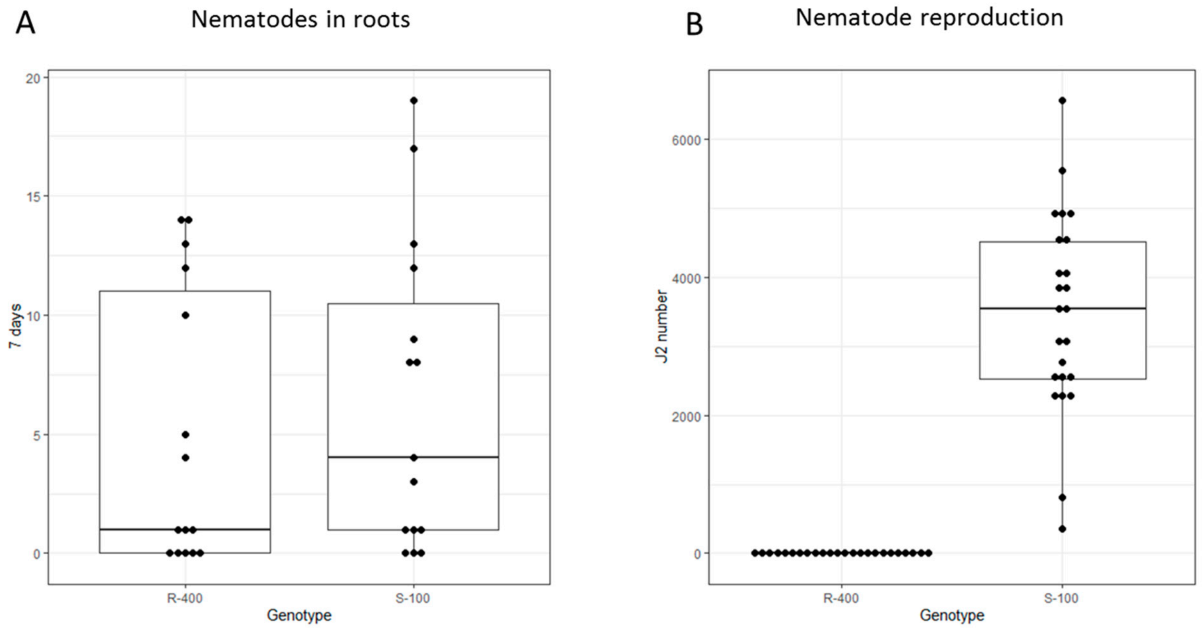

Supplement: Supplementary file 1 [file pathogens-09-00644-s001.zip › Figure S1.pdf]
