# Supplementary material for: Analyses of the Root-Knot Nematode (Meloidogyne graminicola) Transcriptome during Host Infection Highlight Specific Gene Expression Profiling in Resistant Rice Plants
Source: Pathogens. 2020 Aug 8;9(8):644. doi: 10.3390/pathogens9080644 (PMC7460394; doi:10.3390/pathogens9080644)

Figure S2. **Expression profiles of *M. graminicola* genes determined by quantitative RT-PCR experiments.** Y-axes represent the relative fold change of gene expression levels at 2, 4 and 8 dpi in susceptible (Nip2, Nip4, Nip8) or resistant (Tog2, Tog4, Tog8) rice plants compared to the pre-parasitic J2 stage (pre-J2). Three independent biological assays were analysed (dark grey = replicate A, grey = replicate B, both used for RNA-seq libraries; light grey = replicate C). *Mg449* and *Mg693* share homologies with CWDE genes, *Mg1702* and *Mg2129* with neuropeptide genes, *Mg707* and *Mg11937* with VAP genes, *Mg4965* with CLE-like genes and other *Mg* genes are candidate effector and pioneer genes.

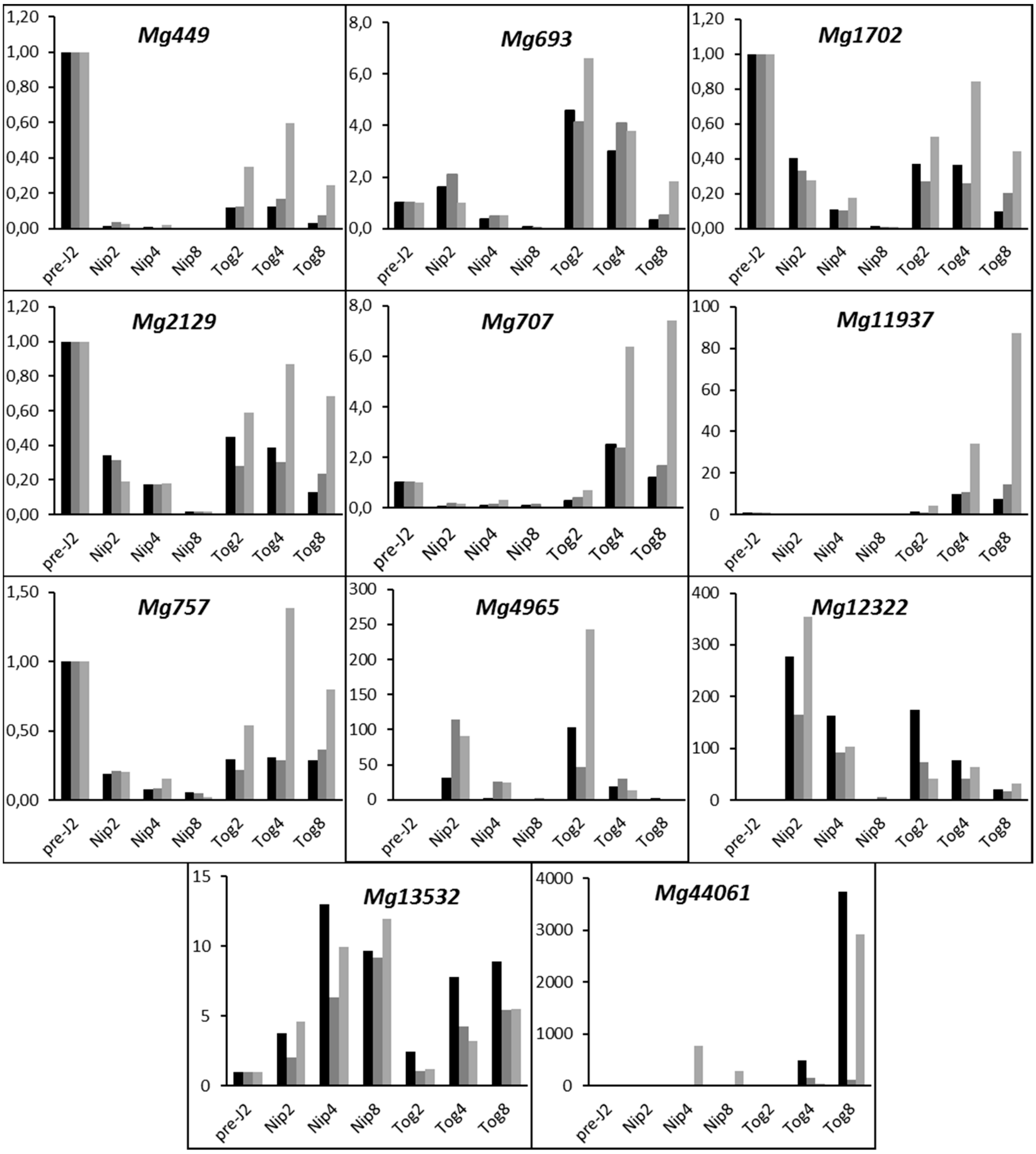

Supplement: Supplementary file 1 [file pathogens-09-00644-s001.zip › Figure S2.pdf]
