# Supplementary material for: Analyses of the Root-Knot Nematode (Meloidogyne graminicola) Transcriptome during Host Infection Highlight Specific Gene Expression Profiling in Resistant Rice Plants
Source: Pathogens. 2020 Aug 8;9(8):644. doi: 10.3390/pathogens9080644 (PMC7460394; doi:10.3390/pathogens9080644)
Supplement: Supplementary file 1 [file pathogens-09-00644-s001.zip › Table S1.pdf]

Table S1. Summary statistics for the *M. graminicola* Mg_v2 transcriptome

***M. graminicola* Mg_v2 transcriptome statistics**		
Number of sequences		44 137
Number of loci		29 825
Number of loci with one transcript		19 769
Number of loci with 2 or more transcripts		10 056
Mean size (bp)		1 212
Median size (bp)		753
Number of transcripts < 0.5 kb		16 131
Number of transcripts > 1 kb		18 014

**Blast results (sequences with at least one hit)**	**Number**	**%**
blastn Mg_v2 against *M. graminicola* (10.895 CDS, [24])	22 489	50.95
blastn Mg_v2 against *M. graminicola* (4.304 contigs, [24])	25 392	57.53
blastx Mg_v2 against *M. incognita* v3 (43.718 proteins, [25])	27 340	61.94
blastx Mg_v2 against *M. hapla* (14.420 proteins, [27])	25 398	57.54
blastx Mg_v2 against *C. elegans* (26.150 proteins, [28])	22 382	50.71
blastx Mg_v2 against nr (Swissprot)	23 596	53.46
Mg_v2 sequences with no hit	7095	16.07
blastn Mg_*M. graminicola* (10.895 CDS, [24]) against Mg_v2	9 511	87.30
tblastn Mg_*M. incognita* v3 (43.718 proteins, [25]) against Mg_v2	34 057	77.90

**Library name and number of read pairs**	**mapped reads on Mg_v2**	**%**
J2A (34 024 074)	47 769 368	70.20
J2B (31 774 556)	43 336 949	68.19
Nip2A (32 263 267)	374 340	0.58
Nip2B (36 026 736)	294 708	0.41
Nip4A (30 335 264)	1 082 875	1.78
Nip4B (35 280 340)	860 458	1.22
Nip8A (35 015 567)	6 792 880	9.70
Nip8B (36 776 218)	3 407 061	4.63
Tog2A (33 556 047)	635 336	0.95
Tog2B (44 016 997)	669 538	0.76
Tog4A (41 467 625)	1 436 869	1.73
Tog4B (40 418 360)	1 393 648	1.72
Tog8A (36 199 434)	3 144 207	4.34
Tog8B (32 759 506)	2 499 263	3.81
